# Impact of Black Soldier Fly Larvae Oil on Immunometabolic Processes

**DOI:** 10.3390/ijms26104855

**Published:** 2025-05-19

**Authors:** Hadas Inbart Richter, Ofer Gover, Amit Hamburg, Keren Bendalak, Tamar Ziv, Betty Schwartz

**Affiliations:** 1 Institute of Biochemistry, Food Science and Nutrition, The School of Nutritional Sciences, The Robert H. Smith Faculty of Agriculture, Food and Environment, The Hebrew University of Jerusalem, Rehovot 7610001, Israel; 2 Smoler Proteomics Center, Technion-Israel Institute of Technology, Haifa 3200003, Israel

**Keywords:** black soldier fly larvae (BSFL), macrophage polarization, cytokines, toll-like receptor (TLR), nuclear factor kappa B (NF-κB), peroxisome proliferator-activated receptor (PPAR), immunometabolism

## Abstract

The oil extract derived from black soldier fly (*Hermetia illucens*) larvae (BSFL) is characterized by a distinctive fatty acid composition and bioactive compounds with demonstrated anti-inflammatory properties, as shown in our previous work. The present study aims to mechanistically explore the immunomodulatory effects of a saponified form of BSFL oil (MBSFL) and its potential interaction with metabolic signaling pathways. Using Pam3CSK4-polarized M1 primary human peripheral blood mononuclear cells (PBMCs), we demonstrate that MBSFL phenotypically suppressed the secretion of pro-inflammatory cytokines TNFα, IL-6, IL-17, and GM-CSF (*p* < 0.01) without altering anti-inflammatory cytokine levels (TGFβ1, IL-13, and IL-4). A phosphoproteomic analysis of Pam3CSK4-stimulated THP-1 macrophages revealed MBSFL-mediated downregulation of CK2 and ERK kinases (*p* < 0.05), key regulators of NF-κB signaling activation. We confirmed that MBSFL directly inhibits NF-κB p65 nuclear translocation (*p* < 0.05), using both immunofluorescence staining and a western blot analysis of nuclear and cytoplasmic fractions. In the context of metabolism, using a luciferase reporter assay, we demonstrate that MBSFL functions as a weak agonist of PPARγ and PPARδ (*p* < 0.05), which are nuclear receptors involved in lipid metabolism and immune regulation. However, subsequent immunoblotting revealed a macrophage polarization-dependent regulation: MBSFL upregulated PPARγ in M0 macrophages but did not prevent its suppression upon Pam3CSK4 stimulation, whereas it specifically enhanced PPARδ expression during M1 polarization (*p* < 0.05). This study provides novel experimental evidence supporting our hypothesis of MBSFL’s role in immunometabolism. We demonstrate for the first time that MBSFL acts as a dual regulator by suppressing NF-κB-mediated inflammation while promoting PPARδ activity—an inverse relationship with potential relevance to immunometabolic disorders.

## 1. Introduction

The oil extract from black soldier fly (*Hermetia illucens*) larvae (BSFL), traditionally used as a commodity in feed applications, such as broiler diets [1], has recently gained attention as a valuable and sustainable ingredient due to its distinctive fatty acid profile, notably comprising 40–50% lauric acid. Additionally, BSFL oil contains immunomodulatory metabolites, including eicosanoids, such as anti-inflammatory lipoxygenase-derived omega-3 and omega-6 oxylipins, CYP450-derived omega-6 epoxy-oxylipins, N-acylamides, and N-acylethanolamines, as well as anti-inflammatory isoprenoids, such as sterols and squalene. This unique composition underlies its demonstrated nutritional [2,3], anti-bacterial [4,5], and, as shown in our previous study, anti-inflammatory activities [6]. The present study aims to elucidate the signaling pathways mediating the anti-inflammatory effects of BSFL oil in vitro.

The innate immune response involves M1 macrophage polarization through toll-like receptor (TLR) signaling, secreting pro-inflammatory mediators, including tumor necrosis factor α (TNFα) and interleukins (ILs), such as IL-6, IL-1β, IL-12, and IL-23, to promote inflammation for effective pathogen clearance. TLR activation triggers downstream signaling, with the nuclear factor kappa B (NF-κB) pathway being the most prominently described in the literature [6]. In the canonical pathway, inhibitor κB kinase (IKK) phosphorylates inhibitor κB (IκB), tagging it for proteasomal degradation. This releases NF-κB p50/p65 dimers from IκB’s cytoplasmic sequestration, resulting in their rapid nuclear translocation [7,8]. Additional NF-κB pathways include the non-canonical activation of p52/RelB dimers via NF-κB-inducing kinase (NIK) [9], and the atypical pathway that relies on sequential p38 and casein kinase II (CK2) phosphorylation of IκB [10,11]. The release of NF-κB dimers is followed by multiple post-translational modification events, among which phosphorylation of p65 is crucial for optimal NF-κB dimer activation [10,12,13]. Subsequent binding to their specific κB sites in the promoter or enhancer regions initiates chromatin remodeling, recruitment of co-activators, and assembly of the basal transcriptional machinery complex [12]. NF-κB signaling is interconnected with parallel pathways, including an interwoven NF-κB/mitogen-activated protein kinase (MAPK) cascade, that synergistically amplifies the inflammatory response upon TLR activation [14]. During the resolution phase, macrophages differentiate into distinct M2 subsets—M2a, M2b, M2c, and M2d—depending on specific stimuli, with potential overlap between these subsets. These macrophages alleviate the detrimental effects of prolonged inflammation through the secretion of anti-inflammatory mediators, including the cytokines IL-10, IL-13, and transforming growth factor Beta 1 (TGFβ1) [15]. Macrophage polarization is regulated by bidirectional interactions between immune cell functions and metabolic pathways; this field is known as immunometabolism. M1 macrophages are characterized by elevated glycolysis and glutamine utilization to support inflammatory responses. In contrast, M2 macrophages preferentially utilize fatty acid oxidation and glutamine metabolism, processes regulated by AMP-activated protein kinase (AMPK) and peroxisome proliferator-activated receptors (PPARs), along with other signaling pathways [16,17]. PPARs are ligand-inducible nuclear receptors comprising three isotypes: PPARα (*NR1C1*), PPARβ/δ (PPARδ; *NR1C2*), and PPARγ (*NR1C3*). Ligand activation of PPARδ promotes an anti-inflammatory M2 phenotype [18], partly through the regulation of genes involved in fatty acid oxidation [19]. However, PPARγ is the primarily expressed PPAR in M2 macrophages [20], reportedly enhancing anti-inflammatory mediators, such as IL-13, IL-4, IL-10, and Arginase (ARG)-1 [21,22]. ARG1 contributes to the M2 anti-inflammatory response by converting arginine into polyamines and prolines, promoting tissue repair, and by indirectly inhibiting cytosolic nitric oxide production from inducible nitric oxide synthase (iNOS). ARG2, an isoform of ARG1, is located in the mitochondria and is implicated in inducing pro-inflammatory cytokine production through mitochondrial reactive oxygen species (ROS) production [23].

Given BSFL oil’s rich composition of lipid-soluble metabolites with anti-inflammatory potential, we sought to investigate its ability to modulate M1 macrophage plasticity across the M1–M2 polarization spectrum and to explore its role in immunometabolism. The objective of this study is therefore to contribute to the development of innovative nutritional therapies that utilize bioactive compounds capable of modulating TLR signaling, thereby advancing the field of immunonutrition.

## 2. Results

### 2.1. Saponified Black Soldier Fly Larvae Oil (MBSFL) Suppresses M1-Associated Pro-Inflammatory Cytokine Secretion Without Altering M2-Associated Cytokines

**Figure 1:**
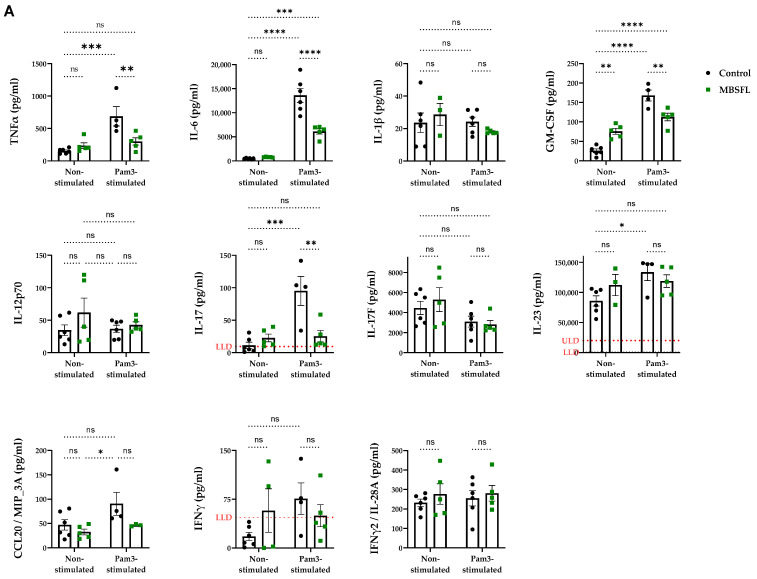

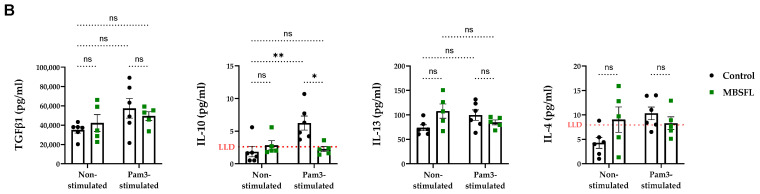
The effect of saponified BSFL oil (MBSFL) on the pro- and anti-inflammatory cytokine characteristics of macrophage polarization. Human primary peripheral blood mononuclear cells (PBMCs) differentiated with 50 ng/mL macrophage colony-stimulating factor (M-CSF) were stimulated with 250 ng/mL Pam3CSK4 (Pam3) for 20 h, in the presence or absence of 600 µM MBSFL. (**A**) Pro-inflammatory and (**B**) anti-inflammatory cytokine secretion levels. Cytokine levels in the supernatants were quantified using a multiplexed sandwich enzyme-linked immunosorbent assay (ELISA)-based quantitative array (*n* = 5–6 ± SEM). Statistical significance: ns = not significant, * *p* < 0.05, ** *p* < 0.01, *** *p* < 0.001, and **** *p* < 0.0001. Results exceeding the detection range are reported relative to the lower limit of detection (LLD) or upper limit of detection (ULD).

To evaluate the potential of saponified BSFL oil (MBSFL) in modulating macrophage plasticity across the M1–M2 polarization spectrum, we investigated its role in regulating pro- and anti-inflammatory cytokine secretion in a model of human primary peripheral blood mononuclear cells (PBMCs) differentiated with macrophage colony-stimulating factor (M-CSF). We assessed MBSFL’s effects under both M0 (unstimulated) and M1 (stimulated with Pam3CSK4 (Pam3) for 20 h) conditions. As shown in Figure 1, activation of TLR2 by Pam3 significantly increased the secretion of pro-inflammatory cytokines, including TNFα, IL-6, IL-17, and granulocyte-macrophage colony-stimulating factor (GM-CSF), as well as the anti-inflammatory cytokine IL-10, all of which were attenuated by MBSFL. Neither Pam3 nor MBSFL affected the secretion levels of the anti-inflammatory cytokines TGFβ1 or IL-13, and the concentrations of IL-4 remained consistently near the lower limit of detection (LLD) throughout the trial. Cell viability remained high across all experimental conditions (Supplementary Figure S1B), confirming that these treatment effects were not due to cytotoxicity.

### 2.2. Phosphoproteomic Analysis Unveils a Role for MBSFL in Chromatin Dynamics to Inhibit Inflammation

**Figure 2:**
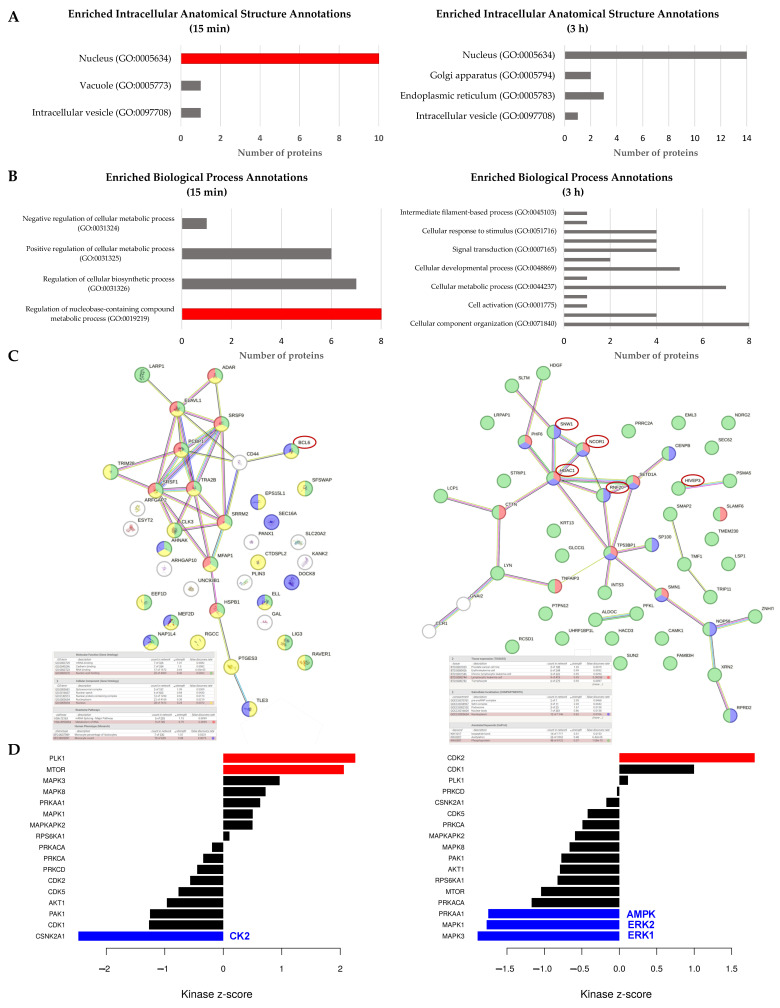
MBSFL supplementation drives dynamic changes in the phosphoproteome of toll-like receptor (TLR)-2-activated macrophages. Differential effects of MBSFL on phosphorylation, 15 min and 3 h following Pam3-stimulation (50 ng/mL) of phorbol 12-myristate-13-acetate (PMA)-primed THP-1 macrophages. (**A**) Enriched intracellular anatomical structure annotations showing differential ratios from cellular components and (**B**) enriched biological process annotations, derived from the Protein ANalysis THrough Evolutionary Relationships (PANTHER) classification system for phospho-proteins identified as differentially expressed by MBSFL supplementation. (**C**) Protein–protein interaction network of differentially phosphorylated proteins. The network was generated using the STRING database. Red circles highlight proteins referenced in the Results. (**D**) Kinase-Substrate Enrichment Analysis (KSEA). The top kinases are depicted using a *p*-value threshold of 0.05, downregulated kinases shown in blue and upregulated kinases shown in red.

To gain insight into the mechanism by which MBSFL antagonizes the inflammatory response, we employed a phosphoproteomic analysis for MBSFL effects in an in vitro model of phorbol 12-myristate-13-acetate (PMA)-primed THP-1 macrophages, stimulated with Pam3 for 15 min and 3 h. The Gene Ontology (GO) enrichment analysis revealed that, 15 min after MBSFL treatment, there was a significant enrichment of phosphorylation events associated with the nucleus (GO:0005634) as well as regulation of nucleobase-containing compound metabolic processes (GO:0019219), collectively suggesting modulation of pathways involved in gene expression and nucleic acid metabolism (Figure 2A,B). The STRING phospho-protein interaction network analysis further elucidated that, at both 15 min and 3 h post-stimulation, MBSFL influenced the phosphorylation status of several key trans-acting factors involved in chromatin dynamics, including the transcriptional co-repressors nuclear receptor corepressor 1 (NCOR1), histone deacetylase 1 (HDAC1), and ring finger protein 20 (RNF20). Additionally, phosphorylation changes were observed in NF-κB cistromic transcriptional co-repressors B-cell lymphoma 6 (BCL6) and ZAS3 (encoded by *HIVEP3*) (Figure 2C). The kinase substrate enrichment analysis (KSEA) revealed that MBSFL treatment led to an increase in the predicted activity of kinases involved in cell proliferation, specifically polo-like kinase 1 (PLK1) and cyclin-dependent kinase 2 (CDK2), at 15 min and 3 h after Pam3 stimulation, respectively. This increase was concomitant with a predicted reduction in the activity of AMPKα1 (encoded by *PRKAA1*), a central regulator of cellular energy sensing and metabolic homeostasis. Moreover, the activities of CK2 (encoded by *CSNK2A1*) and extracellular signal-regulated kinases 1/2 (ERK1/2; encoded by *MAPK3/1*), both crucial for inflammatory responses in M1 macrophages, were decreased in the presence of MBSFL at 15 min and 3 h post-Pam3 stimulation, respectively (Figure 2D). These findings corroborate MBSFL’s anti-inflammatory properties and suggest its involvement in modulating chromatin remodeling dynamics to suppress inflammation.

### 2.3. MBSFL Modulates Anti-Inflammatory Responses Through Suppression of Nuclear Factor Kappa B (NF-κB) p65 Nuclear Translocation

**Figure 3:**
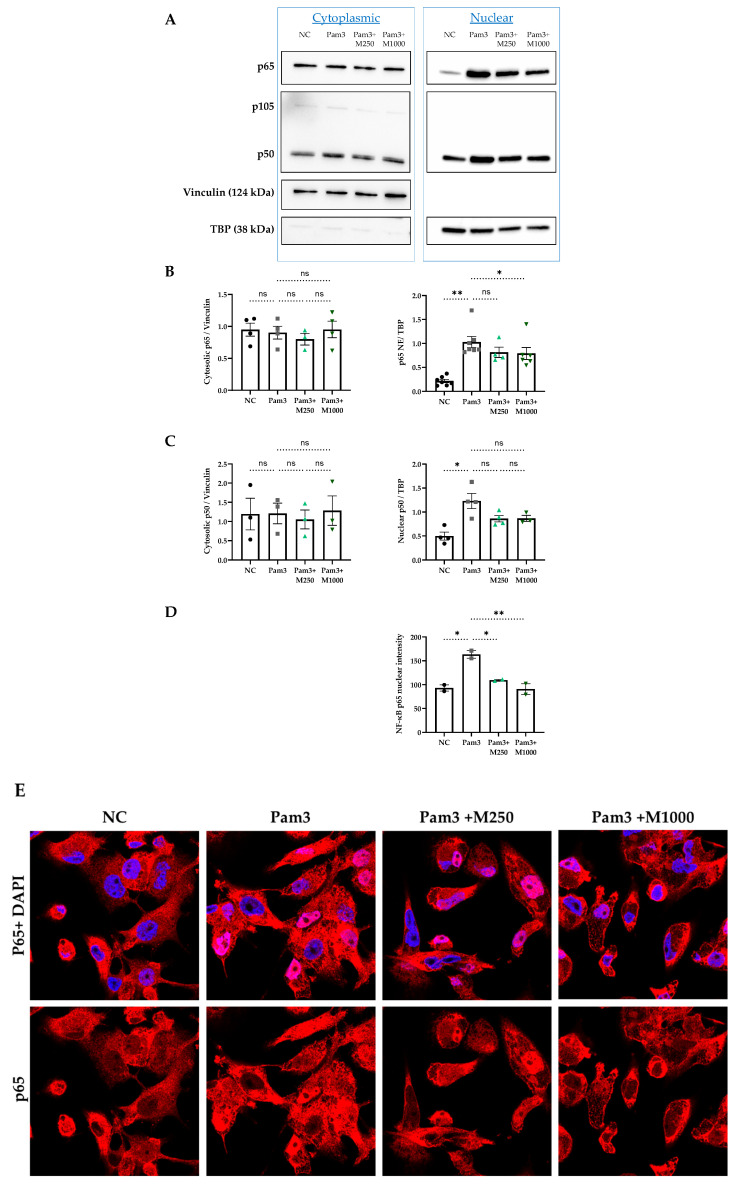
Regulation of nuclear factor kappa B (NF-κB) nuclear translocation by MBSFL in TLR2-activated macrophages. PMA-primed THP-1 macrophages were stimulated with 50 ng/mL Pam3 in the presence or absence of MBSFL at the indicated concentrations for 12 h. Unstimulated cells served as the negative control (NC). (**A**) Subcellular NF-κB p65 and p50 protein levels, detected by western blotting and (**B**,**C**) quantified by densitometry. Vinculin and TBP were used as loading controls for cytoplasmic and nuclear protein fractions, respectively, to ensure equal protein loading and fraction purity (*n* = 3–6 ± SEM). (**D**) Quantification and (**E**) representative confocal images of p65 nuclear immunofluorescence intensity (red), with nuclei counterstained with DAPI (blue). Magnification ×100. Data represent the mean ± SEM from 2 biological repeats, each with 3 technical repeats. Statistical significance: ns = not significant, * *p* < 0.05 and ** *p* < 0.01.

Nuclear translocation of NF-κB dimers is pivotal for driving pro-inflammatory cytokine expression in activated M1 macrophages. To assess the effect of MBSFL on this process, we employed two independent techniques: western blot analysis of subcellular fractions and immunofluorescence staining. Pam3 stimulation for 12 h resulted in significant accumulation of p50 and p65 in the nucleus, while MBSFL treatment reduced p65 nuclear levels in a dose-dependent manner (Figure 3A–E). Cytoplasmic p50 and p65 levels remained unchanged with either Pam3 stimulation or MBSFL supplementation, indicating that the observed effects were due to altered nuclear translocation rather than changes in overall protein expression (Figure 3A–C). The levels of the p50 precursor, p105, were undetectable (Figure 3A).

### 2.4. Role of MBSFL in Immunometabolism via Activation of Peroxisome Proliferator-Activated Receptor (PPAR)-δ

**Figure 4:**
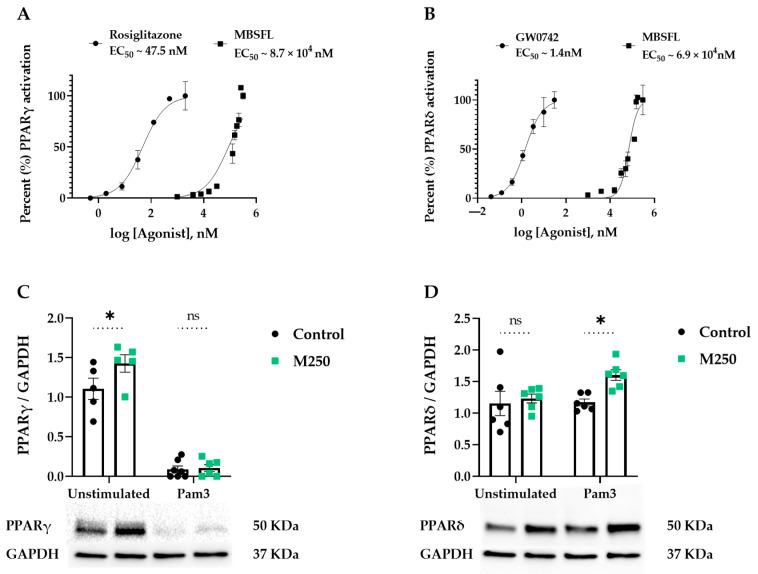

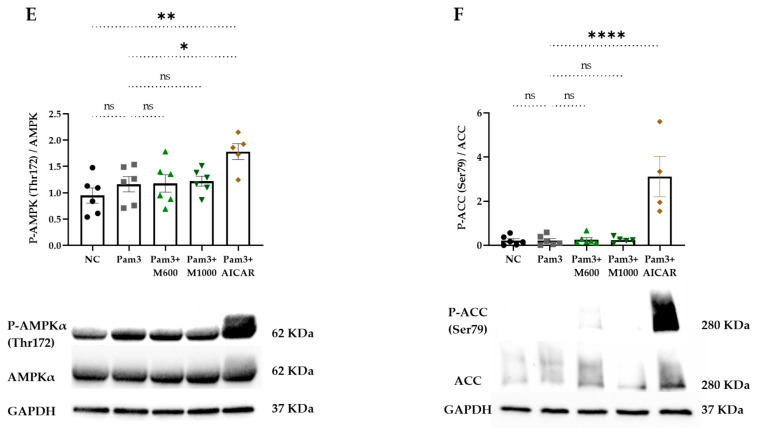
Effect of MBSFL on the activity of metabolic modulators in TLR2-activated macrophages. (**A**,**B**) Dose-response curves of MBSFL oil for peroxisome proliferator-activated receptor (PPAR)-γ and PPARδ agonism, respectively, as measured using a luciferase-based cell assay. Compound concentrations were log-transformed, and relative light units (RLUs) were normalized to the percent activity of the receptor. Non-linear regression analyses were performed to determine half maximal effective concentration (EC_50_) values (*n* = 2 ± SEM). (**C**–**F**) PMA-primed THP-1 macrophages were stimulated with 50 ng/mL Pam3 in the presence or absence of MBSFL at indicated concentrations, and protein levels were determined using western blotting and densitometry: (**C**,**D**) PPARγ and PPARδ protein levels were determined 24 h after stimulation; (**E**,**F**) phosphorylation levels of AMP-activated protein kinase (AMPK) and acetyl CoA carboxylase (ACC) were analyzed 3 h and 22 h after stimulation, respectively. AICAR (0.5 mM) served as a positive control for AMPK activation. Values were normalized to their respective unphosphorylated state or GAPDH (*n* = 4–6 ± SEM). Statistical significance: ns = not significant, * *p* < 0.05, ** *p* < 0.01, and **** *p* < 0.0001.

We next attempted to identify the interplay between inflammation and metabolism by evaluating the effect of MBSFL on key metabolic factors involved in M1 attenuation: PPARs and AMPK. PPAR activation was measured by a luciferase reporter gene assay and protein expression was measured by western blotting. As depicted in Figure 4A,B, MBSFL oil enhanced both PPARγ and PPARδ activation, albeit demonstrating relatively weak agonistic activity, with a half maximal effective concentration (EC_50_) approximately 10^3^–10^4^ times higher relative to the reference agonists. The western blot analysis revealed that MBSFL significantly increased PPARγ protein levels in PMA-primed quiescent THP-1 cells. However, upon Pam3 stimulation for 24 h, PPARγ abundance was nearly abolished, and MBSFL failed to antagonize this effect (Figure 4C). Conversely, MBSFL increased PPARδ abundance upon Pam3 stimulation (Figure 4D). AMPK activity, as was evaluated by its relative phosphorylation levels on Thr172, and the phosphorylation levels of its downstream target, acetyl CoA carboxylase (ACC), was relatively low in all treatment groups, in marked contrast to their increase by AICAR, an AMPK activator reference (Figure 4E,F). These findings suggest that the anti-inflammatory effects of MBSFL are mediated through PPARδ activation.

**Figure 5:**
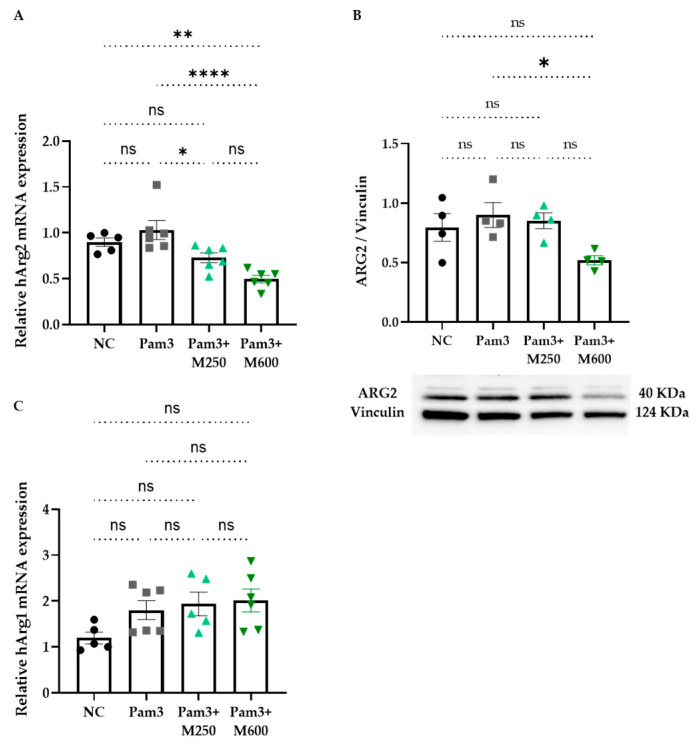
Arginase (ARG) regulation by MBSFL in TLR2-activated macrophages. PMA-primed THP-1 macrophages were left quiescent (NC) or stimulated with 50 ng/mL Pam3 in the presence or absence of MBSFL oil at indicated concentrations. (**A**,**C**) Real-time PCR (qPCR) analysis of *Arg2* and *Arg1* mRNA levels 12 h after stimulation, respectively. Data were normalized to GAPDH; (**B**) ARG2 protein levels 22 h after stimulation, analyzed by western blotting and densitometry, and normalized to vinculin. Data represent the mean ± SEM (*n* = 6), ns = not significant, * *p* < 0.05, ** *p* < 0.01, **** *p* < 0.0001.

We further examined the effect of MBSFL on the expression of ARG1 and ARG2 genes and proteins in PMA-primed THP-1 macrophages at 12 h and 22 h after stimulation with Pam3, respectively. MBSFL supplementation at a concentration of 600 µM resulted in the downregulation of *Arg2* expression, accompanied by a decrease in protein levels (Figure 5A and Figure 5B, respectively). Conversely, *Arg1* mRNA levels were not affected by MBSFL oil in Pam3-stimulated cells (Figure 5C), and protein levels were undetected in any condition tested.

## 3. Discussion

The present investigation sought to elucidate the anti-inflammatory properties of MBSFL and delineate its modulatory effects on cellular signaling cascades within the framework of immunometabolism. Our findings demonstrate that MBSFL effectively alleviates TLR2-activated M1 inflammatory cytokines secretion through a mechanism involving the interplay between immune function and cellular metabolism, via reductions in ERK and CK2 activity and diminished nuclear levels of NF-κB p65, and concomitant with activation of PPARδ, collectively contributing to the modulation of the inflammatory response.

**Figure 6:**
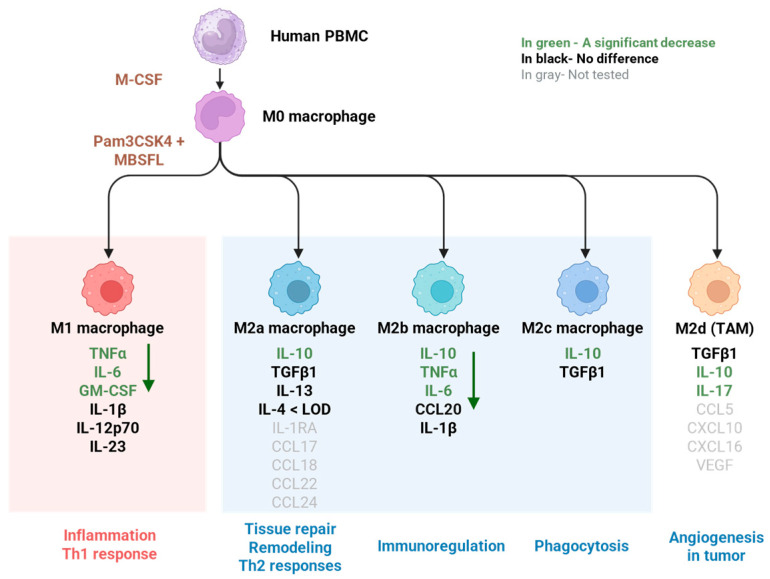
Schematic representation of MBSFL-mediated modulation of cytokine secretion profiles in TLR2-activated, M-CSF-primed PBMCs, elucidating its potential effect on macrophage phenotypic plasticity. The diagram was generated using BioRender.com, with the foundational template adapted from Orekhov et al. [27], and further refined to incorporate study-specific findings on MBSFL’s immunomodulatory effects.

The anti-inflammatory activity of MBSFL is evidenced by its suppression of pro-inflammatory cytokine secretion (TNFα, IL-6, IL-17, and GM-CSF) in Pam3-stimulated human primary PBMCs, which are established markers of TLR2-activated M1 macrophages [24]. Conversely, M2-associated cytokines (TGFβ1, IL-13, and IL-4) remained unaffected by MBSFL supplementation. An exception was IL-10, whose secretion increased following Pam3 stimulation, but was subsequently antagonized by MBSFL. However, although categorized as an anti-inflammatory cytokine, it has been reported that TLR-activated macrophages also secrete IL-10 as part of a delayed negative feedback mechanism to limit inflammation [25], possibly involving the activation of the non-canonical NF-κB p52 protein, which binds to the IL-10 promoter [26]. Therefore, the effect of MBSFL on the complex cytokine profile involved in macrophage plasticity suggests a role in limiting inflammation without promoting a shift toward an M2 phenotype, as illustrated in Figure 6.

To elucidate the molecular underpinnings of MBSFL’s anti-inflammatory action, we leveraged phosphoproteomic approaches in PMA-primed THP-1 macrophages stimulated with Pam3 for 15 min or 3 h. The phosphoprotein dataset demonstrated alterations in nucleic acid metabolism and the phosphorylation status of key transcriptional co-repressors. Notably, this included ZAS3, NCOR1, and BCL6, which have been shown to inhibit NF-κB binding to its DNA motif [28,29,30]. Furthermore, MBSFL modulated the phosphorylation of co-repressors and activators, which, although pleiotropic, also reportedly influence NF-κB-mediated transcriptional activity, such as HDAC1 [31], RNF20 [32], and SNW1 [7]. The KSEA revealed that MBSFL upregulates cell cycle-promoting kinases PLK1 and CDK2, while downregulating CK2 and the MAPK ERK1/2. Since M1 polarization involves cell cycle arrest at the G1 to S transition, and the CDK2-cyclin E complex drives the G1 to S phase transition [33,34], these findings support MBSFL in diminishing an additional aspect of M1 manifestation. Moreover, the CDK2-cyclin E complex has been reported to suppress NF-κB-mediated transcriptional activation [35]. CK2 and ERK play multifaceted roles in macrophage polarization and function. In M0 macrophages, they promote cell proliferation through ERK-mediated activation of CK2 [36]. In contrast, during M1 polarization, CK2 and ERK activate pro-inflammatory signaling pathways, and are greatly involved in phosphorylation events in the NF-κB pathway. CK2 activates NF-kB via the atypical pathway by direct phosphorylation and subsequent degradation of IkBα, followed by p65 phosphorylation at Ser276 by protein kinase A (PKA). Interestingly, this site can also be phosphorylated by mitogen- and stress-activated kinase 1 (MSK1), a downstream effector of ERK1, within the nucleus. Additionally, CK2 directly phosphorylates and activates cytoplasmic p65 on Ser529 [10]. Therefore, the predicted decrease in CK2 and ERK by MBSFL further corroborates its capacity to impinge upon macrophage M1 polarization. Collectively, these correlative phosphoproteomic findings, supported by secondary literature, suggest mechanistic hypotheses warranting further study through targeted assays in future research. A direct inhibitory effect on NF-κB nuclear translocation was demonstrated through complementary methodological approaches, including immunofluorescence staining and a western blot analysis of subcellular fractions. Both techniques confirmed the attenuation of p65 nuclear levels by MBSFL, supporting its role in modulating NF-κB signaling.

**Figure 7:**
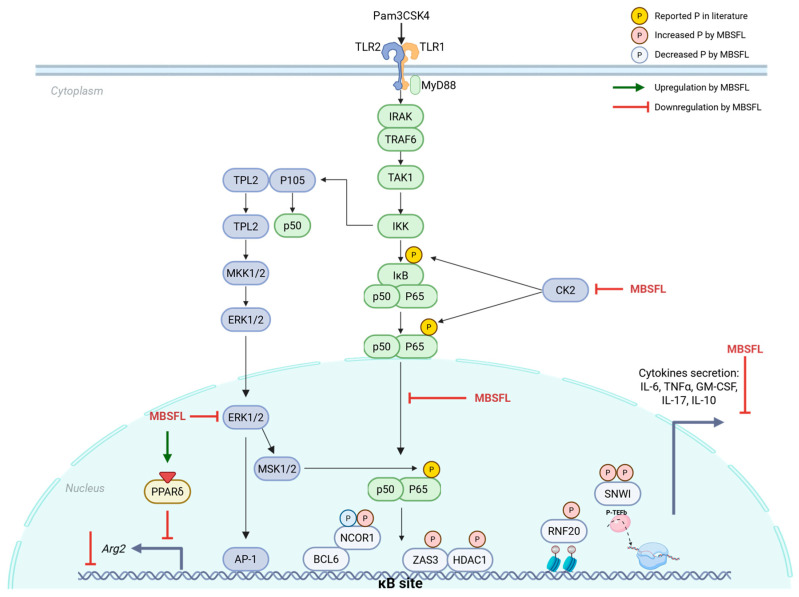
Illustration depicting the potential immunometabolic regulation of MBSFL on pro-inflammatory responses in M1 macrophages. The diagram highlights the modulation of key signaling factors by MBSFL, indicated by red or green arrows. TLR2 signaling in macrophages activates the NF-κB pathway through the degradation of inhibitor κB (IκB), which releases p50/p65 dimers to translocate to the nucleus and activate the transcription of pro-inflammatory genes. MBSFL inhibits NF-κB p65 nuclear translocation. Furthermore, MBSFL reduces the activity of casein kinase II (CK2), a kinase that facilitates p50/p65 activation via the atypical pathway, by phosphorylating IκB at Ser293 and p65 at Ser529. MBSFL also diminishes extracellular signal-regulated kinases (ERK) activity, which mediates p65 phosphorylation and activation at Ser276 within the nucleus. Additionally, MBSFL acts as an activator of PPARδ, which reportedly antagonize both ERK activation and p50/p65 nuclear translocation. This cascade of effects ultimately leads to the attenuation of M1 macrophage pro-inflammatory responses, as evidenced by a reduction in the secretion of pro-inflammatory cytokines. Additional signaling molecules depicted include: myeloid differentiation primary response 88 (MyD88), interleukin-1 receptor-associated kinase (IRAK), TNF receptor-associated factor 6 (TRAF6), transforming growth factor beta-activated kinase 1 (TAK1), IκB kinase (IKK), B-cell lymphoma 6 (BCL6), nuclear receptor corepressor 1 (NCOR1), histone deacetylase 1 (HDAC1), zinc finger and SCAN domain-containing protein 3 (ZAS3), SNW domain-containing protein 1 (SNWI), ring finger protein 20 (RNF20), tumor progression locus 2 (TPL2), mitogen-activated protein kinase kinase 1/2 (MKK1/2), mitogen- and stress-activated protein kinase 1/2 (MSK1/2) and activator protein 1 (AP-1). Created with BioRender.com.

The immunometabolic interplay underlying MBSFL’s anti-inflammatory effects was suggested in our previous study to involve PPAR activation. The RNA sequencing network analysis predicted an increase in PPARδ direct target genes by MBSFL treatment, and LC-MS profiling identified PPARγ agonists DiHOMEs and 13S-HOTrE as the predominant MBSFL eicosanoid components [6]. In this study, we show that MBSFL exerts an agonistic activity against both PPARδ and PPARγ, although with lower potency compared to the reference agonist and limited statistical power. Remarkably, its effect on their protein levels was found to be dependent on the macrophage polarization state. While MBSFL upregulated PPARγ in M0 macrophages but did not prevent its abolishment upon Pam3 stimulation, it increased PPARδ abundance specifically in Pam3-stimulated macrophages, suggesting that MBSFL activation of PPARδ, and not of PPARγ, may limit the inflammatory response. In support with these observations, cytosolic ARG1, a canonical M2 polarization marker directly regulated by PPARγ [37], remained absent in our in vitro model irrespective of MBSFL treatment. AMPK, an M2 metabolic inducer, was likewise not activated by MBSFL. These results align with MBSFL’s inability to activate anti-inflammatory cytokine secretion, collectively suggesting a diminished propensity to promote M2 polarization. In contrast, *Arg2*, an inverse PPARδ transcriptional target [19], demonstrated significant MBSFL-mediated downregulation, as evidenced by reduced mRNA and protein levels. Moreover, the inhibition of ERK has been reported to reduce *Arg2* promoter activity in lipopolysaccharide (LPS)-activated RAW 264.7 macrophages [38]. Collectively, these findings suggest that the anti-inflammatory effects of MBSFL are mediated through PPARδ activation and concurrent inhibition of the CK2-ERK-NF-κB signaling axes, as depicted in Figure 7. An antagonistic relationship between liganded PPARδ and NF-κB p65 has been reported, with several mechanisms of action proposed. These include a direct trans-repressing interaction in the nucleus [39], or indirectly, via downregulation of ERK1/2 phosphorylation to inhibit LPS-induced IL-6 expression in 3T3-L1 adipocytes [40]. In macrophages, ligand-activated PPARδ reportedly suppresses NF-κB signaling by releasing BCL6 from inhibition, enabling BCL6-mediated recruitment of NCOR1 to NF-κB target gene promoters, thereby repressing their transcription [41]. The potential involvement of MBSFL in the crosstalk between NF-κB and PPARδ presents an exciting avenue for investigation in future studies. While this study presents novel preliminary evidence suggesting a mechanistic role for MBSFL in attenuating inflammatory responses, it is limited by its reliance primarily on in vitro models and indicative analyses. Nonetheless, supporting in vivo data obtained from a DSS-induced colitis model, as published in our previous study, provide an important foundation. Future research should focus on targeted validation of the proposed pathways and expansion to additional in vivo models to establish causality and assess clinical relevance.

## 4. Materials and Methods

### 4.1. Saponified BSFL Oil (MBSFL)

BSFL oil extract primarily composed of triglycerides (Entoprotech Ltd., Caesarea, Israel) was saponified, i.e., reacted with potassium hydroxide to produce glycerol and lauric acid salts, as described in a published international patent application WO2020234884A1 [42]. The saponified BSFL oil (MBSFL) was further solubilized in BSA (Sigma-Aldrich, Burlington, MA, USA, endotoxin-free fraction V, FA-poor, catalog no. 126579) to a concentration of 100 mM at 40 °C for 30 min (stock solution). Fresh stock solutions were prepared 24 h before each experiment.

### 4.2. Cell Culture

PBMCs isolation and culture. This study was approved by the Committee for the Use of Human Subjects in Research at The Robert H. Smith Faculty of Agriculture, Food and Environment, Israel (approval code AGHS/May-1.24). All procedures were conducted by students certified by the Collaborative Institutional Training Initiative (CITI), and written informed consent was obtained from all participants. Human peripheral blood samples were collected from nine healthy volunteers (aged 25–50 years, both sexes, not taking medications) using heparin-coated tubes (Danyel Biotech, Rehovot, Israel). PBMC-derived macrophages were prepared as previously described [43] with slight modifications. PBMCs were isolated using Ficoll–Paque density gradient media (Cytiva, Marlborough, MA, USA, catalog no. 17544202), washed with phosphate-buffered saline (PBS; SARTORIUS, Göttingen, Germany), and resuspended in Roswell Park Memorial Institute (RPMI) 1640 culture medium (SARTORIUS, Göttingen, Germany) containing 5% human serum (freshly isolated from a pool of nine healthy volunteers for each experiment using VACUETTE tubes, Danyel Biotech, Israel) and 1% penicillin–streptomycin (Sigma-Aldrich Chemical Co., Burlington, MA, USA). Cells were seeded in 24-well plates (Costar TC-Treated, Corning, New York, NY, USA) at a density of 1 × 10^6^ cells/mL (600 µL per well). After 3 h, non-adherent cells (lymphocytes) were removed by washing with RPMI 1640. Adherent cells (monocytes) were differentiated for 6 days in culture medium supplemented with 20 ng/mL M-CSF (GIBCO, Oxford, UK). The medium was replaced once on day 4. On day 7, the medium was changed to culture medium without M-CSF, allowing a 24 h recovery period before commencing experiments.

Human myeloid leukemia cell line THP-1 (ATCC, Manassas, VA, USA; catalog no. TIB-202™) was maintained in RPMI 1640 medium supplemented with 10% heat-inactivated fetal bovine serum (HI-FBS; Gibco, UK, catalog no. 10082147), 1% penicillin–streptomycin, and 1% sodium pyruvate (both from Sigma-Aldrich Chemical Co., USA). For experimental procedures, cells were seeded in 6-well plates (Costar TC-Treated, Corning, USA) at a density of 1 × 10^6^ cells/well and differentiated using 10 ng/mL PMA (Sigma-Aldrich Chemical Co., USA) for 72 h. Following differentiation, cells were washed with PMA-free medium before experimental treatments. All cultures were maintained at 37 °C in a humidified atmosphere containing 5% CO_2_.

### 4.3. Cell Treatment

Cells were treated with MBSFL at concentrations ranging from 250 to 1000 µM, with vehicle controls receiving BSA alone. Vehicle-treated controls were included and analyzed in all downstream assays alongside treatment groups. 5-aminoimidazole-4-carboxamide ribonucleotide (AICAR; Sigma-Aldrich Chemical Co., Burlington, MA, USA, catalog no. A9978) was treated at 0.5 mM in positive control samples for P-AMPK and P-ACC activation. One hour after treatment, cells were stimulated with the synthetic triacylated lipopeptide Pam3CSK4 (Pam3; InvivoGen, San Diego, CA, USA, catalog code: tlrl-pms) at 250 ng/mL for PBMC cultures or 50 ng/mL for THP-1 cells. Following the stimulation period, supernatants and cells were harvested for subsequent analysis.

### 4.4. Cell Viability Assay

Cell viability was evaluated by the thiazolyl blue tetrazolium bromide (MTT) test. Cells were seeded and treated as described above. At the end of the experiment, supernatants were removed and fresh high glucose DMEM without phenol red (Sartorius, Israel) containing 0.25 mg/mL of MTT (Thermo Fisher Scientific Inc., San Diego, CA, USA, catalog no. L11939) was added to each well for 2 h. Finally, insoluble formazan crystals were dissolved in 100 µL dimethyl sulfoxide (DMSO; Bio-Lab Ltd., Jerusalem, Israel) for 15 min, and the absorbance was measured at 570 nm with a plate reader (Infinite M Plex, Tecan Trading AG, Switzerland). The viability was determined as the percentage of viable cells in treated cultures compared to the percentage of the control group.

### 4.5. Multiplexed Sandwich ELISA-Based Quantitative Array

Cytokine analysis was performed using Quanti-body^®^ Human TH17 Array 1 quantitative kit (RayBiotech, Peachtree Corners, GA, USA, catalog no. QAH-TH17-1). Supernatants from PBMC treatments were diluted 1:1 with the sample diluent and analyzed according to the manufacturer’s protocol. Each sample was arrayed in quadruplicate.

### 4.6. Phosphoproteomic Analysis

Protein lysates were prepared via trypsin digestion, followed by isotype labeling as described in Supplementary Method S1. A portion of the lysates was reserved for total proteomics analysis, while the remaining sample was enriched for phosphopeptides using TiO2. Peptides were subsequently analyzed by LC-MS/MS on an Exploris 480 mass spectrometer (Thermo Fisher Scientific, San Diego, CA, USA), coupled with a Vanquish capillary HPLC system (Thermo Fisher Scientific, San Diego, CA, USA), as detailed in Supplementary Method S1. Mass spectrometry data were processed using Proteome Discoverer 2.4 (Thermo Fisher Scientific, San Diego, CA, USA) with the Sequest search engine, searching against the human proteome (UniProt, January 2024). Details of modifications and search parameters are provided in Supplementary Method S1. Quantification was performed using the Dimethylation2plex method and normalized to the total peptide amount. Peptide-level false discovery rates (FDRs) were controlled at 1% using the target-decoy strategy. Bioinformatics tools employed include the STRING database [44] and the KSEA web-based tool [45,46].

### 4.7. Immunofluorescence Staining and Confocal Microscopy

THP-1 cells were seeded as described above, with coverslips inserted into each well. Following treatment, cells were washed once with Hanks′ balanced salt solution (HBSS) containing Ca^2+^/Mg^2+^ (Sartorius, Israel) and fixed with 4% paraformaldehyde (Bar Naor, Israel) for 30 min at room temperature. After fixation, cells were washed three times with PBS and permeabilized with Perm/Wash Buffer (BD Biosciences, USA) for 45 min at room temperature. The cells were then incubated overnight at 4 °C with anti-p65 antibody conjugated to Alexa Fluor 647 (Santa Cruz Biotechnology, Delaware Avenue, CA, USA, catalog no. sc-8008-AF647) diluted in blocking buffer (CAS-Block, Thermo Fisher Scientific Inc., San Diego, CA, USA). The following day, cells were washed three times with PBS, stained with 300 nM DAPI (Invitrogen, Carlsbad, CA, USA) for 5 min, washed three times with PBST (PBS with 0.2% Tween 20, Sigma-Aldrich Chemical Co., Burlington, MA, USA), and mounted onto coverslips with mounting media (Enzo Biochem, Farmingdale, NY, USA). Images were acquired from three distinct, non-overlapping areas per slide (technical triplicates) using a Leica Stellaris 5 FLIM STED confocal microscope. The nucleus was manually outlined, and the average intensities of p65 expression within the nucleus were quantified using ImageJ software (version 1.54f; National Institutes of Health -NIH, USA).

### 4.8. Protein Extraction and Quantification

Total protein extraction was performed by washing cells once with cold PBS and lysing them for 30 min on ice in radioimmunoprecipitation (RIPA) lysis buffer (Santa Cruz Biotechnology, Inc., Dallas, TX, USA, catalog no. sc-24948) supplemented with 1% (*v*/*v*) protease and phosphatase inhibitor cocktail (Merck KGaA, Darmstadt, Germany, catalog no. PPC1010). Lysates were vortexed at 10 min intervals during incubation. Following lysis, samples were centrifuged at 14,000 g for 10 min at 4 °C, and the supernatant was collected and stored at−80 °C. Nuclear and cytoplasmic fractions were isolated using the nuclear and cytoplasmic extraction reagents kit (NE-PER; Thermo Fisher Scientific Inc., San Diego, CA, USA, catalog no. 78833) according to the manufacturer’s protocol. Protein concentrations were determined in duplicate using the Pierce BCA Protein Assay Kit (Thermo Scientific, San Diego, CA, USA, catalog no. 23225) following the manufacturer’s instructions.

### 4.9. Western Blot Analysis

Equal amounts of protein were separated on 4–15% gradient SDS-PAGE gels (Bio-Rad, San Francisco, CA, USA catalog no. 4561084) and transferred to polyvinylidene fluoride (PVDF) membranes (Bio-Rad, San Francisco, CA, USA, catalog no. 1704156). Membranes were blocked with 5% Difco skim milk (BD Biosciences, Bedford, MA, USA) in TBST for 1 h at room temperature. Primary antibody incubation was performed overnight at 4 °C on a shaker using the following antibodies from Cell Signaling Technology, Danvers, MA, USA: NF-κB p65 (1:1000; catalog no. 8242S), NF-κB1 p105/p50 (1:1000; catalog no. 13586S), PPARγ (1:1000; catalog no. 2435), PPARδ (1:1000; catalog no. 74076), P-AMPK (Thr172) (1:800; catalog no. 2535), AMPK (1:1000; catalog no. 2532), P-ACC (Ser79) (1:800; catalog no. 3661), ACC (1:1000; catalog no. 3662), ARG1 (1:1000; catalog no. 93668S), ARG2 (1:1000; catalog no. 55003S), vinculin (1:1000; catalog no. 13901S), and GAPDH (1:1000; catalog no. 5174S). TBP antibody was obtained from Abcam (1:2000; catalog no. ab818). After washing three times with TBST, membranes were incubated for 1 h with anti-rabbit IgG, HRP-linked antibody (1:2000; catalog no. 7074), except for TBP, which was followed by incubation with anti-mouse IgG, HRP-linked antibody (1:10,000; Jackson ImmunoResearch, West Grove, PA, USA, catalog no. 715035150). Protein bands were visualized using Amersham enhanced chemiluminescence (ECL) reagent (Cytiva, MA, USA, catalog no. 29018903). Quantification of labeled proteins was performed using an Image Lab system (Bio-Rad, USA). Relative expression levels of whole cell lysates and cytoplasmic extracts were normalized to GAPDH or vinculin, while nuclear extracts were normalized to TBP.

### 4.10. Human PPARs Reporter Assay System

PPARγ and PPARδ transcriptional activities were assessed using luciferase reporter assays (INDIGO Biosciences, cat# IB00101 and IB00121). Reporter cells were seeded in 96-well plates with compound-screening medium (CSM) supplemented with MBSFL at concentrations ranging from 1 to 300 µM, or with reference agonists (rosiglitazone for PPARγ, GW0742 for PPARδ) at varying concentrations as per the manufacturer’s protocol, or vehicle (0.1% BSA). Each concentration was tested in two replicates. After a 23 h incubation at 37 °C in a 5% CO_2_ humidified incubator, the treatment medium was removed, and luciferase detection reagent was added. Luminescence was measured by the above plate reader. Dose-response curves and EC_50_ values were generated with GraphPad Prism version 9.0 (GraphPad, San Diego, CA, USA), using the non-linear regression analyses.

### 4.11. Real-Time PCR (qPCR)

Total RNA was isolated from treated THP-1 cells using Nucleospin RNA II kit (Macherey-Nagel, Dueren, Germany) and 1 µg was reversed transcribed with qScript^®^ cDNA Synthesis Kit (Quantabio, Beverly, MA, USA). qPCR amplification was performed in duplicate for each sample using QuantStudio 1 system (Applied Biosystems, Waltham, MA, USA), with Fast SYBR™ Green Master Mix (Applied Biosystems, Waltham, MA, USA). Primer-BLAST (www.ncbi.nlm.nih.gov/tools/primer-blast, accessed on 13 May 2025) was used to design specific primers for *hGAPDH*. Previously described qPCR primer sequences were used for *hArg1* and *hArg2* [47]. The primers used are listed in Supplementary Table S1. All results were normalized to expression of *hGAPDH* gene. The relative expression levels were calculated using the 2^−ΔΔCT^ relative quantification method.

### 4.12. Statistical Analysis

Data were presented as means ± SEM. The statistical differences between groups were determined using the one-way or two-way analysis of variance for multiple comparisons followed by Tukey’s multiple comparisons test in GraphPad Prism version 9.0 (GraphPad, USA), unless stated otherwise. Differences were considered as significant at *p* < 0.05.

## 5. Conclusions

Our study elucidates the immunomodulatory effects of MBSFL on macrophage-mediated pro-inflammatory responses. We demonstrate that MBSFL attenuates the coordinated TLR-activated signaling cascade in macrophages by suppressing the secretion of Pam3-induced pro-inflammatory cytokines. This modulation occurs through several interconnected signaling pathways, including downregulation of CK2 and ERK activity, diminished NF-κB nuclear translocation, and concomitant activation of PPARδ. Collectively, these findings underscore MBSFL’s role in immunometabolism and provide a foundation for further research. Incorporating functional assays in future investigations will be critical to substantiate the biological significance of these molecular alterations and to elucidate their potential therapeutic implications in inflammatory disorders.

## Data Availability

Data is contained within the article and Supplementary Materials.

## Supplementary Materials

The following supporting information can be downloaded at https://www.mdpi.com/article/10.3390/ijms26104855/s1.

## Abbreviations

The following abbreviations are used in this manuscript:

ACC	Acetyl CoA Carboxylase
AICAR	5-Aminoimidazole-4-carboxamide Ribonucleotide
AMPK	AMP-Activated Protein Kinase
ARG	Arginase
BCL6	B-Cell Lymphoma 6
BSA	Bovine Serum Albumin
BSFL	Black Soldier Fly Larvae
CDK2	Cyclin-Dependent Kinase 2
CITI	Collaborative Institutional Training Initiative
CK2	Casein Kinase II
CSM	Compound-Screening Medium
DiHOMEs	Di-Hydroxy-Octadecadienoic Acids
DMSO	Dimethyl Sulfoxide
EC_50_	Half Maximal Effective Concentration
ELISA	Enzyme-Linked Immunosorbent Assay
ERK	Extracellular Signal-Regulated Kinases
FDRs	Peptide-Level False Discovery Rates
GAPDH	Glyceraldehyde-3-Phosphate Dehydrogenase.
GM-CSF	Granulocyte-Macrophage Colony-Stimulating Factor
h	Hour
HBSS	Hanks′ Balanced Salt Solution
HDAC1	Histone Deacetylase 1
IKK	Inhibitor κB Kinase
IL	Interleukin
iNOS	Inducible Nitric Oxide Synthase
IκB	Inhibitor κB
KSEA	Kinase Substrate Enrichment Analysis
LC-MS	Liquid Chromatography-Mass Spectrometry
LLD	Lower Limit of Detection
LPS	Lipopolysaccharide
MAPK	Mitogen-Activated Protein Kinase
MBSFL	Saponified BSFL Oil
M-CSF	Macrophage Colony-Stimulating Factor
min	Minutes
MSK1	Mitogen- and Stress-activated Kinase 1
MTT	Thiazolyl Blue Tetrazolium Bromide (3-(4,5-dimethylthiazol-2-yl)-2,5-diphenyltetrazolium bromide)
MyD88	Myeloid Differentiation Primary Response 88
NCOR1	Nuclear Receptor Corepressor 1
NF-κB	Nuclear Factor Kappa B
NIK	NF-κB-inducing Kinase
Pam3	Pam3CSK4 (Pam3CysSerLys4)
PAMPs	Pathogen-Associated Molecular Patterns
PANTHER	Protein ANalysis THrough Evolutionary Relationships
PBMCs	Peripheral Blood Mononuclear Cells
PBS	Phosphate-Buffered Saline
PKA	Protein Kinase A
PLK1	Polo-Like Kinase 1
PMA	Phorbol 12-Myristate-13-Acetate
PPARs	Peroxisome Proliferator-Activated Receptors
PPRE	Peroxisome Proliferator Response Element
PVDF	Polyvinylidene Fluoride
qPCR	Real-time PCR
RIPA	Radioimmunoprecipitation Assay
RLU	Relative Light Units
RNF20	Ring Finger Protein 20
ROS	Reactive Oxygen Species
RPMI 1640	Roswell Park Memorial Institute 1640
SDS-PAGE	Sodium Dodecyl Sulfate Polyacrylamide Gel Electrophoresis
Ser	Serine
TAK1	TGF-beta Activated Kinase 1
TBP	TATA-binding protein
TGFβ1	Transforming Growth Factor Beta 1
Thr	Threonine
TLR	Toll-like Receptor
TNFα	Tumor Necrosis Factor Alpha
ULD	Upper Limit of Detection
13S-HOTrE	3(S)-Hydroxyoctadecatrienoic acid
