# Supplementary material for: Impact of Black Soldier Fly Larvae Oil on Immunometabolic Processes
Source: Int J Mol Sci. 2025 May 19;26(10):4855. doi: 10.3390/ijms26104855 (PMC12112032; doi:10.3390/ijms26104855)
Supplement: Supplementary file 1 [file ijms-26-04855-s001.zip › ijms-3587613-supplementary.pdf]

Article

Impact of Black Soldier Fly Larvae Oil on Immunometabolic Processes

Hadas Richter [1], Ofer Gover [1], Amit Hamburg [1], Keren Bendalak[2], Tamar Ziv[2] and Betty Schwartz [1,*]

Supplementary Information

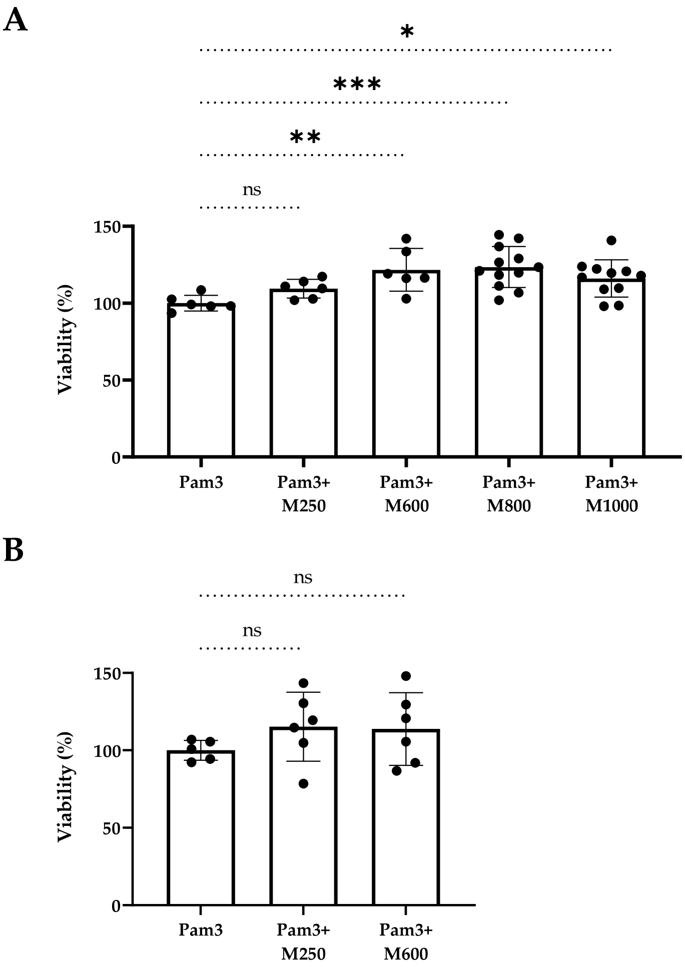

Supplementary Figure S1. Dose-dependent effect of MBSFL on cell viability. (**A**) PMA-primed THP-1 cells and; (**B**) primary peripheral blood mononuclear cells (PBMCs) were stimulated with Pam3CSK4 (Pam3; 50 ng/mL for THP-1 cells and 400 ng/mL for PBMCs) for 24 hours in the presence of MBSFL (M) at the indicated concentrations (μM). Cell viability was assessed using the MTT assay. Data are presented as the mean ± SEM of 5–8 samples, expressed as percentages relative to the positive control (Pam3). Statistical significance is indicated as follows: ns, not significant; *$p < 0.05$; **$p < 0.01$; ***$p < 0.001$.

Supplementary Table S1. Primers used in real time PCR

Target (Gene fragment)	Sequence (5′-3′)	Sequence (3′-5′)
hArg1	ACAAAACAGGGCTACTCTCAGG	CGAGCAAGTCCGAAACAAGC
hArg2	TCAGTGCTGCGGATCATGT	CACTCCTTT TCTTTTCTGCCCTT
hGAPDH	TCACCAGGGCTGCTTTTAAC	GACAAGCTTCCCGTTCTCAG

Supplementary Method S1. Phosphoproteomic analysis

Proteolysis

The protein samples were brought to 8.5M Urea, 100mM ammonium bicarbonate and 10mM DTT, sonicated twice (90%, 10-10, 5′) and centrifuged 10000g 10′. Protein amount was estimated using Bradford readings. The samples were reduced (60ºC for 30 min), modified with 35.2mM iodoacetamide in 100mM ammonium bicarbonate (room temperature for 30 min in the dark) and digested in 1.5M Urea, 17.6mM ammonium bicarbonate with modified trypsin (Promega), overnight at 37°C in a 1:50 (M/M) enzyme-to-substrate ratio. An additional second digestion with Trypsin was done for 4 h at 37°C in a 1:100 (M/M) enzyme-to-substrate ratio. The tryptic peptides were desalted using Oasis HLB extraction cartridges (Waters Corporation, Massachusetts, USA), dried and resuspended in 50mM HEPES at pH 8. Peptides were labelled via reductive methylation with excess of either d0,12C-formaldehyde (HCOH, 4% in water, 20 μL) or d2,13C-formaldehyde (D13COD, 4% in water, 20 μL). Pairs of heavy and light labelled samples were mixed and desalted using Oasis HLB extraction cartridges. A few micrograms were saved for a total proteomics analysis, and the rest was enriched for phospho-peptides using TiO2.

Mass spectrometry analysis

The resulted peptides were analyzed by LC-MS/MS using an Exploris 480 mass spectrometer (Thermo Fisher Scientific Inc., Massachusetts, USA) fitted with a capillary HPLC (Vanquish, Thermo Fisher Scientific Inc., Massachusetts, USA). The peptides were loaded in solvent A (0.1% formic acid in water) on a homemade capillary column (30 cm, 75-micron ID) packed with Reprosil C18-Aqua (Dr. Maisch GmbH, Germany). The peptides mixture was resolved with a 6 to 34% linear gradient of solvent B (80% acetonitrile with 0.1% formic acid) for 180 min followed by a 0.1 min increase of 34 to 99% and 11 minutes at 99% solvent B at flow rates of 0.15 μl/ min. Mass spectrometry was performed in a positive mode (m/z 350–1200, resolution 120,000 for MS1 and 15,000 for MS2), using repetitively full MS scan followed by high collision dissociation (HCD, at 27 normalized collision energy) of the 30 most dominant ions (>1 charges) selected from the first MS scan. A dynamic exclusion list was enabled with an exclusion duration of 30 s.

Data analysis

The mass spectrometry data was analyzed using Proteome Discoverer 2.4 (Thermo Fisher Scientific Inc., Massachusetts, USA) using Sequest search engine, searching against the human proteome from the Uniprot database (82697 entries, downloaded- January 2024) with mass tolerance of 20 ppm for the precursor masses and 0.02 Da for the fragment ions. Oxidation on methionine, phosphorylation on serine, threonine and tyrosine, and protein N-terminus acetylation were accepted as variable modifications. Carbamidomethyl on cysteine, and either heavy dimethylation or light dimethylation on lysine and peptide n-termini were accepted as static modifications. Minimal peptide length was set to six amino acids and a maximum of two miscleavages was allowed. The data was quantified by Dimethylation2plex method using the same software. Normalization was done using Total Peptide Amount. Peptide- level false discovery rates (FDRs) were filtered to 1% using the target-decoy strategy.
